# P-glycoprotein (Mdr1a/1b) and breast cancer resistance protein (Bcrp) decrease the uptake of hydrophobic alkyl triphenylphosphonium cations by the brain

**DOI:** 10.1016/j.bbagen.2013.02.005

**Published:** 2013-06

**Authors:** Carolyn M. Porteous, David K. Menon, Franklin I. Aigbirhio, Robin A.J. Smith, Michael P. Murphy

**Affiliations:** a Department of Chemistry, University of Otago, P.O. Box 56, Dunedin 9054, New Zealand; b Department of Biochemistry, University of Otago, P.O. Box 56, Dunedin 9054, New Zealand; c Division of Anaesthesia, University of Cambridge, Box 93, Addenbrooke's Hospital, Hills Road, Cambridge CB2 0QQ, UK; d Wolfson Brain Imaging Centre, Department of Clinical Neurosciences, University of Cambridge, Addenbrooke's Hospital, Hills Road, Cambridge CB2 0QQ, UK; e MRC Mitochondrial Biology Unit, Hills Road, Cambridge, CB2 0XY, UK

**Keywords:** ABC proteins, ATP binding cassette proteins, BBB, blood–brain barrier, Bcrp, breast cancer resistance protein, CsA, cyclosporin A, IP, intra peritoneal, IV, intra venous, Mdr1, multi drug resistance 1, MitoF, 11-fluoroundecyltriphenylphosphonium mesylate, MitoQ, [10-(4,5-dimethoxy-2-methyl-3,6-dioxo-1,4-cyclohexadien-1-yl)decyl]triphenylphosphonium mesylate, MPTP, 1-methyl-4-phenyl-1,2,3,6-tetrahydropyridine, TPB, tetraphenylborate, TPP, triphenylphosphonium cation, ROS, reactive oxygen species, TPMP, methyltriphenylphosphonium, Mitochondria, Lipophilic cation, Blood–brain barrier, ABC transporters, MitoQ

## Abstract

**Background:**

Mitochondrial dysfunction contributes to degenerative neurological disorders, consequently there is a need for mitochondria-targeted therapies that are effective within the brain. One approach to deliver pharmacophores is by conjugation to the lipophilic triphenylphosphonium (TPP) cation that accumulates in mitochondria driven by the membrane potential. While this approach has delivered TPP-conjugated compounds to the brain, the amounts taken up are lower than by other organs.

**Methods:**

To discover why uptake of hydrophobic TPP compounds by the brain is relatively poor, we assessed the role of the P-glycoprotein (Mdr1a/b) and breast cancer resistance protein (Bcrp) ATP binding cassette (ABC) transporters, which drive the efflux of lipophilic compounds from the brain thereby restricting the uptake of lipophilic drugs. We used a triple transgenic mouse model lacking two isoforms of P-glycoprotein (Mdr1a/1b) and the Bcrp.

**Results:**

There was a significant increase in the uptake into the brain of two hydrophobic TPP compounds, MitoQ and MitoF, in the triple transgenics following intra venous (IV) administration compared to control mice. Greater amounts of the hydrophobic TPP compounds were also retained in the liver of transgenic mice compared to controls. The uptake into the heart, white fat, muscle and kidneys was comparable between the transgenic mice and controls.

**Conclusion:**

Efflux of hydrophobic TPP compounds by ABC transporters contributes to their lowered uptake into the brain and liver.

**General significance:**

These findings suggest that strategies to bypass ABC transporters in the BBB will enhance delivery of mitochondria-targeted antioxidants, probes and pharmacophores to the brain.

## Highlights

► Brain accumulation of triphenylphosphonium cations is decreased by ABC transporters. ► ABC-transporter inactivation increases brain uptake of triphenylphosphonium cations. ► Bypassing ABC transporters may increase the effectiveness of mitochondrial therapies.

## 1 Introduction

Mitochondrial dysfunction contributes to a wide range of degenerative neurological disorders and to acute brain damage [1], [2], [3], [4]. Consequently there is considerable interest in developing therapies that decrease mitochondrial damage and preserve organelle function [1], [3]. Mitochondria-targeted therapies based on lipophilic alkyl triphenylphosphonium (TPP) cations have been developed and show promise in vivo and in human trials [1], [5], [6]. These TPP lipophilic cations pass directly through phospholipid bilayers due to their large hydrophobic surface area lowering the activation energy for uptake [5], [7], [8], while their positive charge causes their accumulation several-hundred fold within mitochondria inside cells, driven by the plasma and mitochondrial membrane potentials [5], [7]. These properties have been used to deliver a range of TPP cations selectively to mitochondria within cells, including: antioxidants [5], [9], [10], [11], thiol reagents [12], [13], spin traps [14], [15], [16], [17], fluorescent reactive oxygen species (ROS) probes [18], [19], [20], [21], toxins [22], [23], DNA alkylating agents [24] and nitric oxide donors [25]. One of these compounds, the mitochondria-targeted antioxidant mitoquinone (MitoQ), has shown efficacy in a number of animal models of pathologies [26], [27], [28], [29], [30], [31], [32]. Furthermore, MitoQ has been used in humans and shown to be safe during long-term oral administration [33] and to be effective at decreasing liver damage in a preliminary phase II trial in hepatitis C patients [34]. Therefore mitochondria-targeted bioactive molecules based on TPP compounds have potential as pharmaceuticals.

A number of animal studies have investigated the distribution of TPP compounds in vivo and these investigations have shown that the uptake of TPP compounds into most tissues was rapid and extensive following long-term oral administration or acute intravenous (IV) or intraperitoneal (IP) delivery [32], [35], [36]. However, while the TPP compounds were taken up into the brain, the extent of uptake was significantly less than into other tissues [32], [35], [36]. Supporting the uptake of TPP compounds by the brain, long-term administration of MitoQ by intraperitoneal injection leads to protection against brain mitochondrial damage in the MPTP animal model of Parkinson's disease [37]. Furthermore, oral delivery of MitoQ to the triple transgenic Alzheimer's disease model also protected against oxidative damage and memory impairment [38]. Therefore while TPP compounds are taken up into the brain this uptake is significantly less than by other organs [35], [36]. The rate and extent of uptake of TPP compounds into cells and tissues is greatly enhanced by increasing the hydrophobicity of the molecule [36], [39], [40]. Increasing the hydrophobicity of TPP compounds by incorporating a 10–11 methylene carbon chain enhances the rate of uptake by lowering the activation energy for movement across the plasma membrane and increases the extent of accumulation within mitochondria by increasing adsorption to the matrix-facing surface of the inner membrane [36], [39], [40]. However, even for hydrophobic TPP cations such as MitoQ and MitoF uptake by the brain was considerably lower than for other organs [36]. This decreased uptake into the brain limits the potency of mitochondria-targeted therapies and the usefulness of probes in the brain. Therefore we set out to understand why the uptake of hydrophobic TPP compounds by the brain is less than for other organs.

A major limitation to the uptake of lipophilic compounds into the brain is the blood–brain barrier (BBB) [41], [42]. The BBB comprises the tightly sealed endothelial cells that form the lumen surface of the capillaries perfusing the brain [41], [42]. These endothelial cells contain tight junctions, have minimal pinocytosis and very few cell fenestrations so that they seal the brain capillaries [41], [42], consequently compounds that enter the brain have to pass through the endothelial cells [41], [42]. Restricting this passage are a series of ATP-binding cassette (ABC) transporters that line the lumenal plasma membrane of the endothelial cells and which pump lipophilic compounds out of the cells, thereby limiting their uptake into the brain [41]. The action of ABC-transporters in the BBB is a major factor limiting the delivery of lipophilic drugs to the brain [42].

The archetypal ABC transporter involved in the BBB lipophilic compound efflux is P-glycoprotein (P-gp, multi drug resistance 1 (Mdr1) protein and in humans as ABCB1), which uses ATP to drive the efflux of lipophilic compounds across the plasma membrane and out of cells [43]. The breast cancer resistance protein (Bcrp, ABCG2) performs a similar function in the BBB [41]. Progress on the structure and mechanism of P-gp suggests that BBB ABC-transporters act as “hydrophobic vacuum cleaners” through a binding site within the membrane that allows a wide range of lipophilic compounds to enter the protein from the plasma membrane, followed by efflux from the cell upon ATP hydrolysis [41], [43], [44], [45]. P-gp is known to actively excrete lipophilic cations [41]. Furthermore, the uptake of MitoQ into cells [46], [47] and its excretion into the bile from the liver in vivo are impeded by the action of P-gp [48]. Together these data implicate the activity of ABC-transporters in the BBB as likely contributors to the relatively poor uptake of lipophilic TPP cations into the brain.

**Fig. 1:**
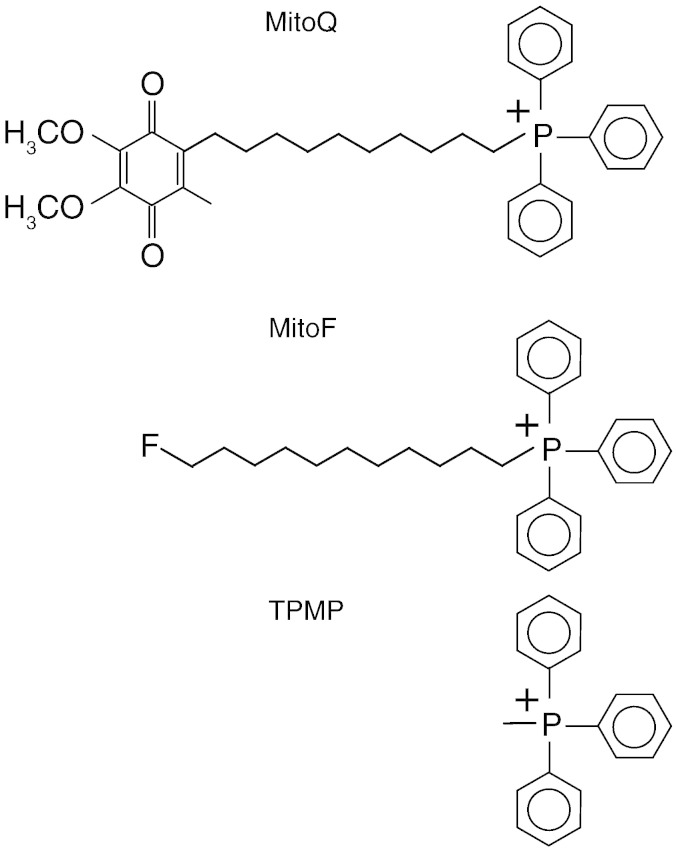
Structures of the TPP cations investigated.

Here we set out to test the hypothesis that the lower uptake of hydrophobic TPP compounds into the brain is due to their excretion from BBB endothelial cells by ABC-transporters such as P-gp. To assess this we measured the acute uptake of the hydrophobic TPP cations MitoQ and MitoF, and the more polar TPP cation methyltriphenylphosphonium (TPMP) (Fig. 1) into the brains of mice lacking the major ABC-transporters. In mice there are two isoforms of P-gp, Mdr1a and Mdr1b (also known as ABCB1a/1b) and a mouse model in which both of these are deleted along with Brcp1 (ABCG2) has been developed [49]. The Mdr1a/1b (−/−)(−/−) Brcp1 (−/−) mouse model has been used to assess the contribution of BBB ABC-transporters to exclusion of lipophilic compounds from the brain [50]. We found that the uptake of MitoQ and MitoF into the brain was far greater in mice lacking these three ABC-transporters, compared with control mice. These findings have important implications for the development of mitochondria-specific therapies and probes.

## 2 Materials and methods

### 2.1 Chemical syntheses

[^3^H] TPMP iodide (60 Ci/mmol) was from American Radiolabeled Chemicals. [^3^H] MitoQ was synthesised and HPLC-purified to > 97% radiopurity, as described [39]. [^3^H] Fluoroundecyltriphenylphosphonium (MitoF) was synthesised and purified as described previously [36]. The published octan-1-ol/PBS partition coefficients are as follows: TPMP, 0.35 [10]; MitoQ, 2760 [51]; MitoF, 740 ± 100 [36].

### 2.2 Administration of compounds to mice

Mice lacking two isoforms of P-gp (ABCB1a and ABCB1b) and Bcrp1 (ABCG2) were created on the Friend leukaemia virus B strain (FVB) background in the laboratory of Prof. Alfred Schinkel of the Netherlands Cancer Institute [49] and female mice (Mdr1a/1b (−/−)(−/−) Brcp1 (−/−)) lacking all three genes were supplied by Taconic Farms (http://www.taconic.com) Model Number 3998-M (FVB.129P2-Abcb1atm1Bor Abcb1btm1Bor Abcg2tm1Ahs N7). Mice were maintained on a 12 h light/dark cycle with ad libitum access to standard lab chow and water for 8–16 weeks prior to experiments.

To assess the effects of co-administration of cyclosporin A (CsA) on the uptake of MitoF, female C57/BL6 mice (~ 20 g) were used. The mice were injected with CsA (Sandimmun, Novartis supplied as 50 mg/mL solution which was diluted 1:4 in sterile PBS and injected 100 μL at 50 mg/kg) IV by tail vein injection, or with PBS carrier, followed 30 min later with [^3^H] MitoF (100 nmol) by IV tail vein injection and 60 min later the mice were killed and the tissues harvested. All procedures were approved by the University of Otago animal ethics committee.

For injection the mice were placed in a restraining tube and injected by the tail vein with 100 nmol [^3^H] compound (~ 400–500 nCi) in 100 μL sterile phosphate buffered saline (PBS) supplemented with 10% DMSO. A sample of the [^3^H] compound solution injected was retained to calculate the specific activity, which was subsequently used to determine the tissue contents of the compound. Two WT mice and two transgenic mice were injected for each compound for each time point. The total number of transgenic mice treated with TPMP, MitoQ or MitoF were, 8, 8 and 10, respectively. The total numbers of WT mice treated with TPMP, MitoQ or MitoF were, 4, 6 and 8, respectively. At the indicated times after injection, the mice were killed by cervical dislocation and a blood sample of ~ 100–200 μL was obtained by cardiac puncture. The organs were then removed, cleared of blood by cutting into pieces and rinsing with saline, transferred to pre-chilled Eppendorf tubes on ice, weighed and stored at − 80 °C until processing. The tissues taken were: heart, kidneys, liver, brain, skeletal muscle (gastrocnemius) and white adipose tissue (subcutaneous). Injection of this amount of MitoQ and other TPP cations was previously shown to be non-toxic [35], as was the case here. The mice were monitored after injection to ensure no pathology or distress.

### 2.3 Extraction of [^3^H] compounds from tissues

**Fig. 2:**
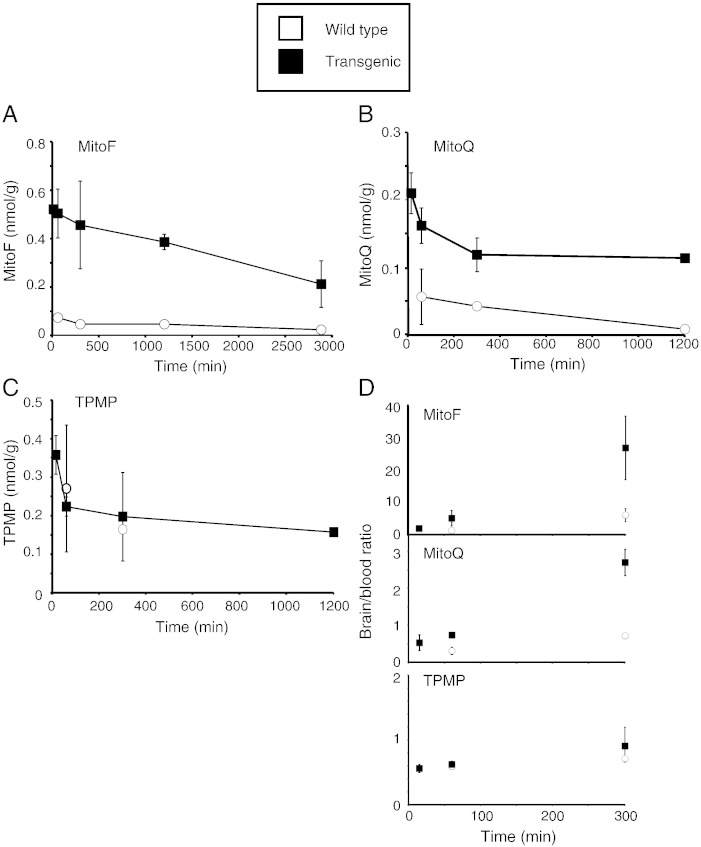
Time course of accumulation of [^3^H] MitoF, [^3^H] MitoQ and [^3^H] TPMP within wild type and transgenic mouse brain following IV injection. Mice were injected with a bolus of 100 nmol [^3^H] TPP compound by IV tail vein injection. At the indicated times the mice were killed and the [^3^H] content in the brain and blood was determined. Data in panels A–C are in nmol/g wet weight tissue and are means ± range for two separate WT mice and two separate transgenic mice per time point. A, MitoF; B, MitoQ; and C, TPMP. In panel D the brain/blood ratios of the three test compounds are shown at 1 h and 5 h post injection. Data are in (nmol compound/g wet weight tissue)/(nmol compound/mL blood).

Tissues were thawed at room temperature and transferred to 50 mL Falcon tubes. Ice-cold methanol (~ 4 °C; 1 mL/100 mg tissue wet weight) was added to the tissue and the tissue homogenised using an Ultra Turrax homogeniser (2 × 30 s on ice). The homogenate was transferred in 1 mL batches to 1.5 mL Eppendorf tubes and centrifuged (10,000 ×*g* for 8 min at 4 °C). The methanol extract was decanted into a 20 mL glass scintillation vial (Wheaton) and the methanol evaporated under a stream of N_2_. Further ice-cold methanol (1 mL/100 mg) was added to the tissue homogenate pellets, vortexed for 1 min, centrifuged as above and the methanol extract decanted to a fresh 20 mL glass scintillation vial and evaporated. This procedure was repeated 3 more times to give 5 extracts per tissue sample. The amount of radioactivity in the fifth extract was always negligible. Scintillant (OptiPhase HiSafe II; 10 mL) was added to each vial, [^3^H] DPM content measured in a scintillation counter (LKB Wallac 1217 Rackbeta) using appropriate quench corrections and the total amount of radioactivity per sample calculated. The specific activity of the injected [^3^H] compound was then used to calculate the tissue content as mol compound/g wet weight tissue. The area under the curve (AUC) for uptake of compounds into the brain were calculated from the brain-time plots in Fig. 2 using the trapezoidal rule.

## 3 Results

### 3.1 Increased uptake of lipophilic TPP compounds into the brain of mice lacking ABC-transporters

To determine whether the presence of ABC-transporters affected the accumulation of TPP cations into the brain we compared the amounts of MitoF, MitoQ and TPMP retained in the brain after administration (Fig. 2). To do this we injected a single bolus of 100 nmol tritiated TPP compound IV into transgenic mice lacking the three ABC transporters and compared the amount of radioactivity present within the brain over 20 h (Fig. 2A–C). For MitoF (Fig. 2A) and MitoQ (Fig. 2B), there was greater uptake into the brain in the transgenic compared to the wild type mice, in contrast, for TPMP there was no difference (Fig. 2C). The area under the curve (AUC) for MitoF accumulation into the brain from 1 to 48 h post injection was 1.9 nmol·h/g wet weight for WT but increased ~ 9-fold to 16.6 nmol·h/g wet weight in the transgenic mice. Similarly, the AUC for MitoQ accumulation into the brain from 1 to 20 h post injection was 0.6 nmol·h/g wet weight for WT but increased ~ 4-fold to 2.3 nmol·h/g wet weight in the transgenic mice. In contrast, the AUC for TPMP accumulation into the brain from 1 to 5 h post injection was 0.87 nmol·h/g wet weight for WT and was unchanged at 0.84 nmol·h/g wet weight in the transgenic mice. The ratios of the compounds in the brain relative to those in the blood 5 h after injection followed the same pattern with increased uptake of MitoF and MitoQ in the transgenic mice, while the distribution of TPMP was the same (Fig. 2D). This confirmed that this difference was due to increased accumulation of MitoF and MitoQ by the brain in the transgenic mice and not due to differences in plasma levels (Fig. 2D). These data indicate that ABC-transporters decrease the extent of accumulation within the brain of the hydrophobic TPP compounds MitoF and MitoQ, but not of the more polar TPMP.

### 3.2 ABC-transporters do not affect uptake of TPP compounds into the heart, kidney or adipose tissue

**Fig. 3:**
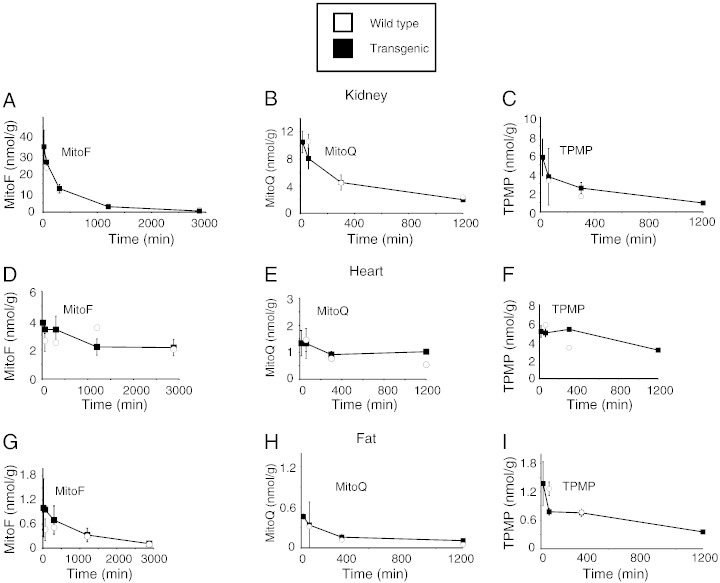
Time course of uptake of [^3^H] MitoF, [^3^H] MitoQ and [^3^H] TPMP into wild type and transgenic mouse tissues following IV injection. Mice were injected with a bolus of 100 nmol [^3^H] TPP compound by IV tail vein injection. At the indicated times the mice were killed and the [^3^H] content in the tissue was determined. Data are in nmol compound/g wet weight tissue and are means ± range for two separate WT mice and two separate transgenic mice per time point. Kidney, panels A–C; Heart, panels D–F; and Fat, panels G–I.

To see if the absence of the ABC-transporters in other organs affected the accumulation of TPP cations by other organs we assessed the heart, kidney and white adipose tissue (Fig. 3A–I). As expected from previous studies [36], there was extensive uptake of MitoF, MitoQ and TPMP by the kidneys, less extensive uptake by the heart and lower uptake by adipose tissue (Fig. 3). There was no difference between the uptake into these three tissues between the wild type and transgenic mice, indicating that the absence of the ABC-transporters does not enhance their acute uptake of TPP cations.

### 3.3 Lack of ABC-transporters increases the accumulation of TPP compounds by the liver

**Fig. 4:**
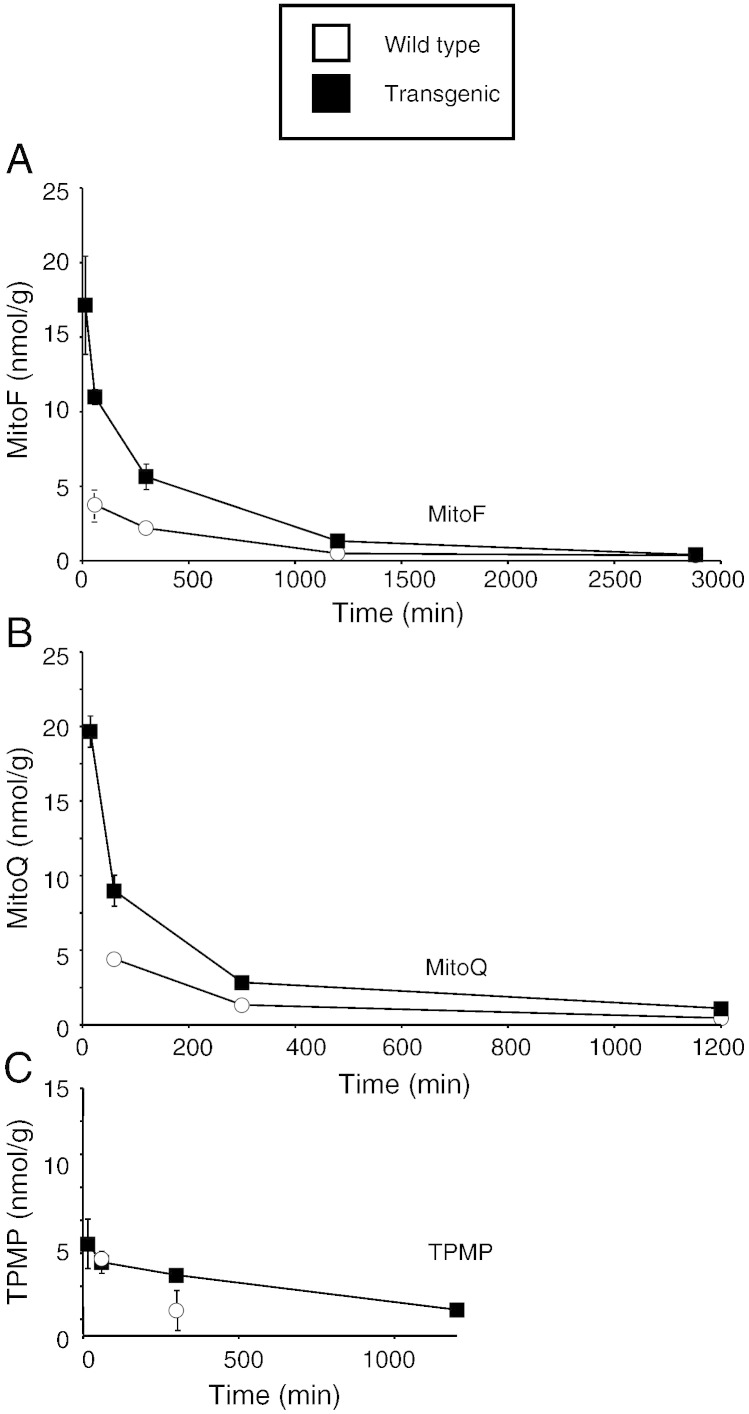
Time course of uptake of [^3^H] MitoF, [^3^H] MitoQ and [^3^H] TPMP into wild type and transgenic mouse liver following IV injection. Mice were injected with a bolus of 100 nmol [^3^H] TPP compound by IV tail vein injection. At the indicated times the mice were killed and the [^3^H] content in the liver was determined. Data are in nmol/g wet weight tissue and are means ± range for two separate WT mice and two separate transgenic mice per time point. MitoF, panel A; MitoQ, panel B; and TPMP, panel C.

In contrast to the heart, kidney and adipose tissue, there was an increase in the uptake of MitoF and MitoQ, but not of TPMP, in the livers of the transgenic mice lacking the three ABC transporters compared to controls (Fig. 4). This suggests that the ABC-transporters are important in the excretion of the more hydrophobic TPP compounds from the liver.

### 3.4 Absence of ABC-transporters does not affect blood levels of TPP compounds

**Fig. 5:**
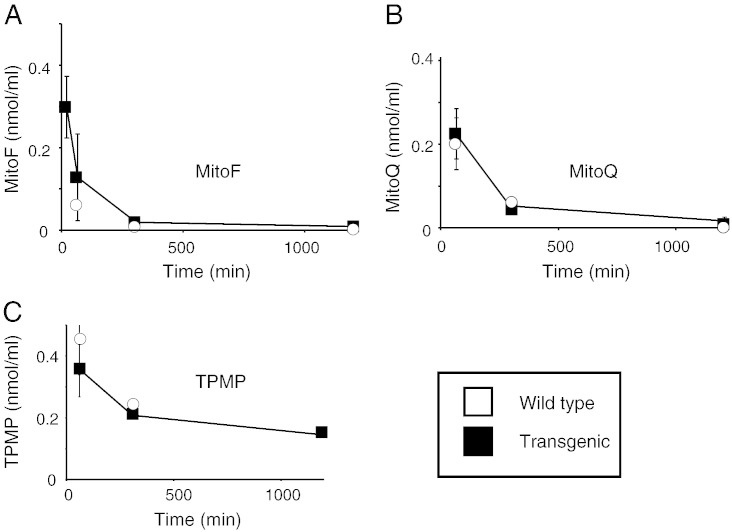
Time course of [^3^H] MitoF, [^3^H] MitoQ and [^3^H] TPMP in blood of wild type and transgenic mouse tissues following IV injection. Mice were injected with a bolus of 100 nmol [^3^H] TPP compound by IV tail vein injection. At the indicated times the mice were killed and the [^3^H] content in the blood were determined (panels A–C). Data are in nmol TPP/mL blood and are means ± range for two separate WT mice and two separate transgenic mice per time point.

To see whether the ABC transporters altered the overall uptake of TPP compounds from the circulation into tissues we next assessed their levels in blood at various times following IV injection (Fig. 5A–C). As expected, all three TPP compounds were rapidly cleared from the blood after IV injection and there were no significant differences in the kinetics of clearance for the three compounds (Fig. 5A–C). Therefore the action of the three ABC transporters does not markedly affect the uptake into most tissues from the plasma of TPP compounds.

### 3.5 Effect of CsA on TPP cation uptake

**Fig. 6:**
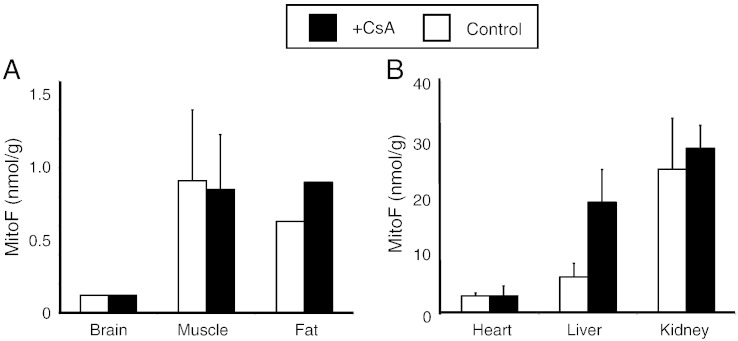
Uptake of [^3^H] MitoF, into wild type mouse tissues following IV injection in the presence of CsA. Mice were pretreated with CsA (50 mg/kg) and 30 min later were injected with a bolus of 100 nmol [^3^H] MitoF by IV tail vein injection. One hour later the mice were killed and the [^3^H] content in the brain, heart, muscle, fat, kidney and liver was determined and compared to that of mice that had not received CsA. Data are in nmol MitoF/g wet weight tissue and are means ± range for two separate WT mice and two separate transgenic mice per time point.

The data so far suggest that the extent of accumulation of hydrophobic TPP cations into the brain and liver is decreased by the action of the three ABC-transporters lacking in the transgenic mice. To see if pharmacological inhibition the ABC-transporters could affect the distribution of the compounds in potentially therapeutically useful ways, we assessed the effect of CsA, an inhibitor of P-gp on the distribution of MitoF (Fig. 6A–B). The administration of CsA prior to the IV injection of [^3^H] MitoF did not affect the uptake of MitoF into the brain, muscle, fat, heart or kidney (Fig. 6A, B). However, CsA did increase uptake into the liver suggesting that co-administration of CsA may increase the content and thus the therapeutic efficacy of lipophilic alkyl TPP molecules in the liver, but not in the brain (Fig. 6B).

## 4 Discussion

The lipophilic TPP cation is widely used to deliver a range of therapeutic and probe molecules such as antioxidants to mitochondria in vivo in animal models and in patients [5], [26], [27], [28], [31], [33], [34]. However, uptake of these compounds into the brain is less than into other organs. To assess why this is so here we have compared the accumulation within the brain between wild type and transgenic mice lacking three of the ABC-transporters that may contribute to exclusion of lipophilic cations in vivo. Here we have shown that the hydrophobic TPP compounds MitoQ and MitoF are accumulated to a greater extent by the brain in vivo in the transgenic mice following IV administration. This suggests that the action of the ABC transporters such as P-gp in the BBB plays a significant role in restricting the accumulation of lipophilic TPP compounds by the brain. Therefore approaches that can bypass this excretion pathway should lead to increased uptake of TPP compounds into the brain and potentially enhance their therapeutic potency for neurological disorders. However, administration of the P-gp inhibitor CsA did not increase the brain uptake of MitoF, even though it altered the liver uptake of MitoF [41], [43], suggesting that other modes to bypass the action of the ABC-transporters in the BBB will be necessary to enhance the delivery of TPP compounds.

There were no major differences between the transgenic and wild type mice in the uptake of MitoQ and MitoF in most other organs, with the exception of the liver, where the uptake of MitoF and MitoQ was greater in transgenic mice. Furthermore, the P-gp inhibitor CsA also increased the levels of MitoF and MitoQ in the liver. Previously it has been shown that a significant proportion of the excretion of MitoQ administered IV was through the liver by the biliary pathway, with 25% of an IV MitoQ dose being excreted in this way within 4 h [52]. This biliary excretion of MitoQ was inhibited by CsA [48]. Together these data are consistent with hydrophobic TPP compounds being excreted by the biliary pathway. The efficacy of inhibition of P-gp and related ABC transporters in the liver by CsA indicates that it is possible to increase the levels of mitochondria-targeted compounds in the liver by inhibiting this pathway.

The uptake of the TPP compounds by the kidney, heart and adipose tissue was the same in the WT and transgenic mice, in contrast to the brain and liver. As the three ABC transporters are relatively poorly expressed in adipose tissue and the heart, the lack of an effect of inactivating the transporters on alkyl TPP cation uptake into these tissues is expected. However there is expression of the ABC transporters in the kidney [53], [54], consequently the absence of a difference in kidney uptake between the WT and transgenic mice for uptake of lipophilic TPP cations is less clear. However, inactivation of ABC transporters frequently has far greater effects on the brain uptake of compounds compared to uptake into the kidney [55], [56]. Therefore, our finding of a difference in uptake between the WT and transgenic mice in the brain but not the kidney is consistent with the literature. The mechanism remains obscure. One possibility is that the dominant pathway(s) for the movement of lipophilic TPP compounds into and out of the kidney cells occurs by pathways distinct from those catalysed by the three ABC transporters. Another possibility is that in the transgenic mice there is a compensatory increase in the activity of other pathways that transport lipophilic cations. This occurs in the Bcrp (−/−) mouse where the compensatory upregulation of two sterol transporter genes, Abcg8 and Abcg5, compensates for the lack of Bcrp [54]. Therefore, we cannot exclude the possibility that in the normal kidney the three ABC transporters investigated here are actually important in the disposition of lipophilic TPP cations but that their loss is compensated for by expression of other transporters. We emphasise that the lack of an effect of alkyl TPP uptake into the kidney in the transgenic mice does not impact on the main conclusion of this paper, which is that the action of these ABC transporters contributes to the exclusion of alkyl TPP cations by the BBB.

There were clear differences in the interaction with the ABC transporters that depended on the hydrophobicity of the tracer alkyl TPP compounds tested. For the two hydrophobic TPP compounds, MitoQ and MitoF, distribution in the brain and liver was dramatically increased by altering the activity of the ABC-transporters. In contrast, the distribution of the more polar TPMP was unaffected by the presence or absence of ABC-transporters. This lack of effect on TPMP is consistent with reported findings for the tetraphenylphosphonium cation, which is of similar hydrophilicity to TPMP: ablation of the two P-gp isoforms Mdr1a and Mdr1b in mice did not increase the uptake of the tetraphenylphosphonium cation across the BBB [57]. One interpretation of these findings is that the increased hydrophobicity of the TPP compounds MitoQ and MitoF increases adsorption to the cytosolic facing surface of the plasma membrane of the endothelial cells and thus increases their access to the hydrophobic binding site of the ABC transporters and thereby enhances their efflux from the cell. For more polar compounds, such as TPMP, their relatively low hydrophobicity makes them poorer substrates for the ABC transporters and consequently we did not see any effect of ablation of the transporters on the distribution of TPMP in the brain or liver. It is also likely that the affinity of the hydrophobic TPP compounds for the ABC transporters is greater than for more hydrophilic compounds. Thus, increased hydrophobicity increases the rate and extent of uptake into tissues in vivo [36], [39]. However, the increased hydrophobicity also increases the adsorption to the plasma membrane and access to, and affinity for, the binding site of the ABC transporters and consequently increased excretion through the liver and decreased uptake through the BBB. Therefore to enhance uptake into the brain it is necessary to both increase hydrophobicity while avoiding excretion by the ABC transporters.

To conclude, there are a number of possible reasons for relative differences in the rate and extent of uptake of a TPP cation into an organ. These include the extent of perfusion of the organ, the possibility that other organs have extracted most of the compound before it arrives and the magnitudes of the plasma and mitochondrial membrane potentials. However it is clear that the action of ABC transporters in the BBB decreases the accumulation of hydrophobic TPP cations into the brain and that overcoming this action of the BBB would significantly improve the targeting of therapeutic molecules and probe molecules to mitochondria in the brain in vivo.
